# LINC01278 Induces Autophagy to Inhibit Tumour Progression by Suppressing the mTOR Signalling Pathway

**DOI:** 10.1155/2023/8994901

**Published:** 2023-01-18

**Authors:** Bo Liu, Xueting Yao, Chaoyang Zhang, Wenzhe Li, Yanan Wang, Qianling Liao, Ziwei Li, Qinying Huang, Yanchen Zhang, Wencan Wu

**Affiliations:** ^1^State Key Laboratory of Ophthalmology, Optometry, And Vision Science, Wenzhou Medical University, Wenzhou, China; ^2^The Eye Hospital, School of Ophthalmology & Optometry, Wenzhou Medical University, Wenzhou, China; ^3^Department of Laboratory Medicine, Longhua Hospital, Shanghai University of Traditional Chinese Medicine, Shanghai, China; ^4^Department of General Surgery, The First Affiliated Hospital of Anhui Medical University, Hefei, China

## Abstract

Uveal melanoma (UM) is an aggressive intraocular malignant tumour that is closely related to autophagic dysfunction. We aimed to identify autophagy-related long noncoding RNAs (lncRNAs) to elucidate the molecular mechanism of UM. Here, we show that LINC01278 is a new potential biomarker with clinical prognostic value in UM through bioinformatics analysis. Application of an autophagy inhibitor (3-MA) and an autophagy agonist (MG-132) indicated that LINC01278 can inhibit UM cell proliferation, migration, and invasion by inducing autophagy. A xenograft nude mouse model was used to examine the tumorigenesis of UM cells in vivo. Mechanistically, LINC01278 can inhibit the mTOR signalling pathway to activate autophagy, as shown by experiments with an mTOR agonist (MHY1485) and mTOR inhibitor (rapamycin) treatment. Our findings indicate that LINC01278 functions as a tumour suppressor by inhibiting the mTOR signalling pathway to induce autophagy. Targeting the LINC01278-mTOR axis might be a novel and promising therapeutic approach for UM.

## 1. Introduction

Uveal melanoma (UM) is the most common primary intraocular malignant tumour among adults, with an incidence rate of 5–10 cases per million people in recent decades [1, 2]. Effective treatments to improve the survival rate of UM are lacking due to the complexity of its molecular mechanisms, such as those closely related to gene mutations, the activation of signalling pathways, and other factors [3–5]. Therefore, exploration of the molecular mechanism of UM to identify diagnostic biomarkers and new effective therapeutic targets is important.

Autophagy is an intracellular catabolic degradation process involving lysosomes that plays a role in adapting to metabolic stress, promoting the renewal of differentiation and development, and preventing genomic damage by degrading abnormal components [6]. Autophagy is involved in various disease processes, including neurodegeneration, infection, cardiovascular disease, and cancer [6–10]. In cancer, autophagy shows inhibitory or activating effects depending on the stage (early or late) and status (nutritional supply, tumour microenvironment, pathogenic factors, and immune system) of cancer development [11, 12]. Recently, the regulation of autophagy in UM has been reported. For example, the autophagy-related protein BECN1 was found to be upregulated in UM, and this upregulation was accompanied by early metastasis and a poor prognosis [13]. The combination of AXIIR overexpression and autophagy inhibitors may enhance the efficacy of UM [14]. Inhibition of mutant GNAQ signalling was shown to induce AMPK-dependent autophagy-mediated death [15]. Importantly, the abnormal expression of long noncoding RNAs (lncRNAs) (CANT1, FTH1P3, MALAT1, and ZNNT1) is involved in the occurrence and development of UM [16–19]. However, the molecular mechanism underlying autophagy-related gene (ARG) regulation in UM still needs further study.

lncRNA transcripts are over 200 nucleotides long and participate in the occurrence and development of many kinds of tumours by abnormally activating signalling pathways [20, 21]. For instance, the lncRNA EMS promotes tumorigenesis by negatively regulating the p53 pathway [22]. miR-423-5p was shown to prevent the MALAT1-mediated proliferation and metastasis of prostate cancer cells [23]. lncRNA HITT inhibited the metastasis of lung adenocarcinoma by reducing Rab5-mediated endocytosis [24]. Moreover, the functional role of lncRNA depends on its intracellular localization. Cytoplasmic lncRNAs might be involved in sponging miRNAs, maintaining the stability of proteins, and modulating signalling pathways. Nuclear lncRNAs might participate in nuclear structure maintenance, chromatin remodelling, and transcriptional regulation [21, 25–28]. Many studies have investigated the role of lncRNAs in UM. For example, downregulation of lncRNA PVT1 could inhibit UM progression by activating the p53 pathway [29]. lncRNA ZNNT1 could induce autophagy to inhibit UM tumorigenesis [19]. Autophagy-related lncRNAs may serve as prognostic markers in UM patients [30]. However, little is known about the mechanism by which lncRNAs modulate the autophagy pathway.

To explore the above scientific problems, we used Pearson's correlation analysis to screen LINC01278, which is closely related to autophagic genes in UM. As a member of the lncRNA family, LINC01278 has been gradually characterized in recent years. However, this molecule acts as a double-edged sword in the formation of tumours. On the one hand, LINC01278 could promote hepatocellular carcinoma metastasis by activating the *β*-catenin/TCF-4-LINC01278-miR-1258-Smad2/3 axis [31] and could promote the progression of osteosarcoma by regulating the miR-133a-3p/PTHR1 signalling pathway [32]. On the other hand, LINC01278 inhibited the progression of papillary thyroid carcinoma by mediating the miR-376c-3p/DNM3 axis [33]. Here, we found that the expression of LINC01278 was significantly decreased in UM cells compared with uveal melanocytes. Moreover, low LINC01278 expression was associated with a poor prognosis in UM. Little is known about the molecular mechanism of LINC01278 in UM. Therefore, we hypothesized that LINC01278 inhibits UM tumour development by promoting autophagy.

In this study, we found that LINC01278 was closely related to UM prognosis through bioinformatics analysis. LINC01278 can inhibit UM formation by promoting autophagy. Mechanistically, LINC01278 can suppress the mTOR signalling pathway to induce autophagy. LINC01278, as a novel prognostic biomarker, may provide a new therapeutic target for the clinical treatment of UM.

## 2. Material and Methods

### 2.1. Analysis of Online Databases

The clinical data and transcriptome sequencing data of 80 UM patients were downloaded from The Cancer Genome Atlas (TCGA) database (https://portal.gdc.cancer.gov/). We obtained a list of 232 human ARGs from the Human Autophagy Database (http://autophagy.lu/clustering/index.html). The methods of data analysis are shown in the literature [34, 35].

### 2.2. Cell Lines and Cell Culture

The OCM1 and MUM-2B cell lines were purchased from the FuHeng Cell Center. All cell lines were examined by short tandem repeat (STR) profiling analysis. The uveal melanocyte cell line (U-94) was obtained from the State Key Laboratory of Ophthalmology, Optometry, and Vision Science, Wenzhou Medical University. All cell lines were cultured in RPMI 1640 medium (Thermo Fisher, C11875500BT) with 10% foetal bovine serum (Lonicera, S711-001S) in an incubator with 5% CO_2_ at 37°C.

### 2.3. Antibodies, Drugs, and Primers for Quantitative Real-Time PCR

For Western blotting, the antibodies were anti-GAPDH (1 : 1000; Bioss, bsm-0978 M), anti-LC3 (1 : 1000; Abcam, ab192890), anti-P62 (1 : 1000; Abcam, ab109012), anti-mTOR (1 : 1000; Abcam, ab134903), anti-CDK1 (1 : 1000; Beyotime, AF1516), anti-CDK2 (1 : 1000; Beyotime, AF1063), and anti-Caspase-3 (1 : 1000; CST, 9662). The drug concentrations were 30 *μ*mol/L for rapamycin (Apexbio, A8167), 30 *μ*mol/L for 3-methyladenine (3-MA, Apexbio, A8353), 40 *μ*mol/L for MG-132 (Apexbio, A2585), and 40 *μ*mol/L for MHY1485 (Apexbio, B5853). The Cytoplasmic and Nuclear RNA Purification Kit was purchased from Norge (21000-50 preps). The primers for Q-PCR were as follows: GAPDH: F: GGAGTCCACTGGCGTCTTCA, R: GTCATGAGTCCTTCCACGATACC; U6: F: TGCTTCGGCAGCACATATAC, R: TCACGAATTTGCGTGTCATC; LINC01278: F: TTGCTCCCAGCATTCCACAA, R: TAATCCTCTTCCAGATGGGG; AP003352.1: F: ATGCGATACCAAGTGACTGACA, R: TATTTGCTGGTTGCCCCTTCA; LINC01637: F: TCCGTGTCCTCCAGACCTTT, R: TGTGTGCATCTCTGCGTTGT; AC036214.2: F: AGCAGCGGGAGATGACTCTA, R: TCTGCACCTACACTGGGTGA; AC090617.5: F: TTGCTTAGCTCCCTGGCAAC, R: CTCCAGTTGTGAGTCCTCGG; UBXN10-AS1: F: GTTGCATAGGTCCCTCGGTT, R: TCCCTTCTGAGACGAGCAGA; SOX1-OT: F: ACCAGAGCCGAGGACTAAAC, R: TTGTTGGTTGCACTACCCCTT.

### 2.4. Plasmids and Small Interfering RNAs (siRNAs)

The human LINC01278 plasmid was purchased from GenePharma (Shanghai, China). The human mTOR plasmid was purchased from the company of Miaoling (Wuhan, China). The LINC01278 lentiviral vector was constructed by GeneChem (Shanghai, China). The siRNA sequences of LINC01278 were synthesized by GenePharma (Shanghai, China) as follows: Si-NC: 5′-UUCUCCGAACGUGUCACGUTT-3′; Si-LINC01278-1: 5′-GGCUUAGAUCUUGUGGCCATT-3′; Si-LINC01278-2: 5′-AACCCAGGGUAACGCUGUCTT-3′.

### 2.5. Cell Proliferation, Migration, and Invasion Assays

For cell proliferation assays, CCK-8 reagent (1 : 11; Beyotime, C0038) was added to each well (96-well plate, 2000 cells/well) for 2 h. The EDU kit was purchased from the company of Beyotime (C0071S). Cell apoptosis was tested by Hoechst's assay (Beyotime, C0003). For cell migration and invasion assays, UM cells (1 × 10^4^ cells/well) were cultured in serum-free RPMI 1640 medium and seeded into the upper chambers (Corning, 3422, 354480). The lower chambers were filled with RPMI 1640 medium with 20% foetal bovine serum. After 24 h, UM cells were fixed in 4% paraformaldehyde (PFA) for 10 min and then stained with crystal violet (Beyotime, C0121) for 10 min.

### 2.6. Xenograft Assays

Four- to six-week-old BALB/c nude mice were purchased from Charles River Laboratories, China, and housed in a specific-pathogen-free (SPF) room at Wenzhou Medical University. UM cells (2 × 10^6^) in 100 *μ*L of PBS were injected subcutaneously into nude mice. The length and width of each tumour were measured every week with a calliper. Tumour volume = (length × width × width)/2. After 4 weeks, the tumours were weighed and photographed.

### 2.7. Statistical Analysis

R software was used to analyse the clinical data and transcriptome sequencing of 80 UM patients, and the chi-square test, Pearson's correlation analysis, univariate and multivariate analyses, or Kaplan-Meier's analysis was adopted. For the experimental data, Student's *t* test was used to analyse two groups of data, and one-way or two-way ANOVA with Bonferroni's post hoc test was applied to more than two groups of data. The data are presented as the mean ± SD with GraphPad Prism 8. *p* < 0.05 was considered statistically significant.

## 3. Results

### 3.1. Prognostic Analysis of Autophagy-Related lncRNAs in UM Patients

**Figure 1:**
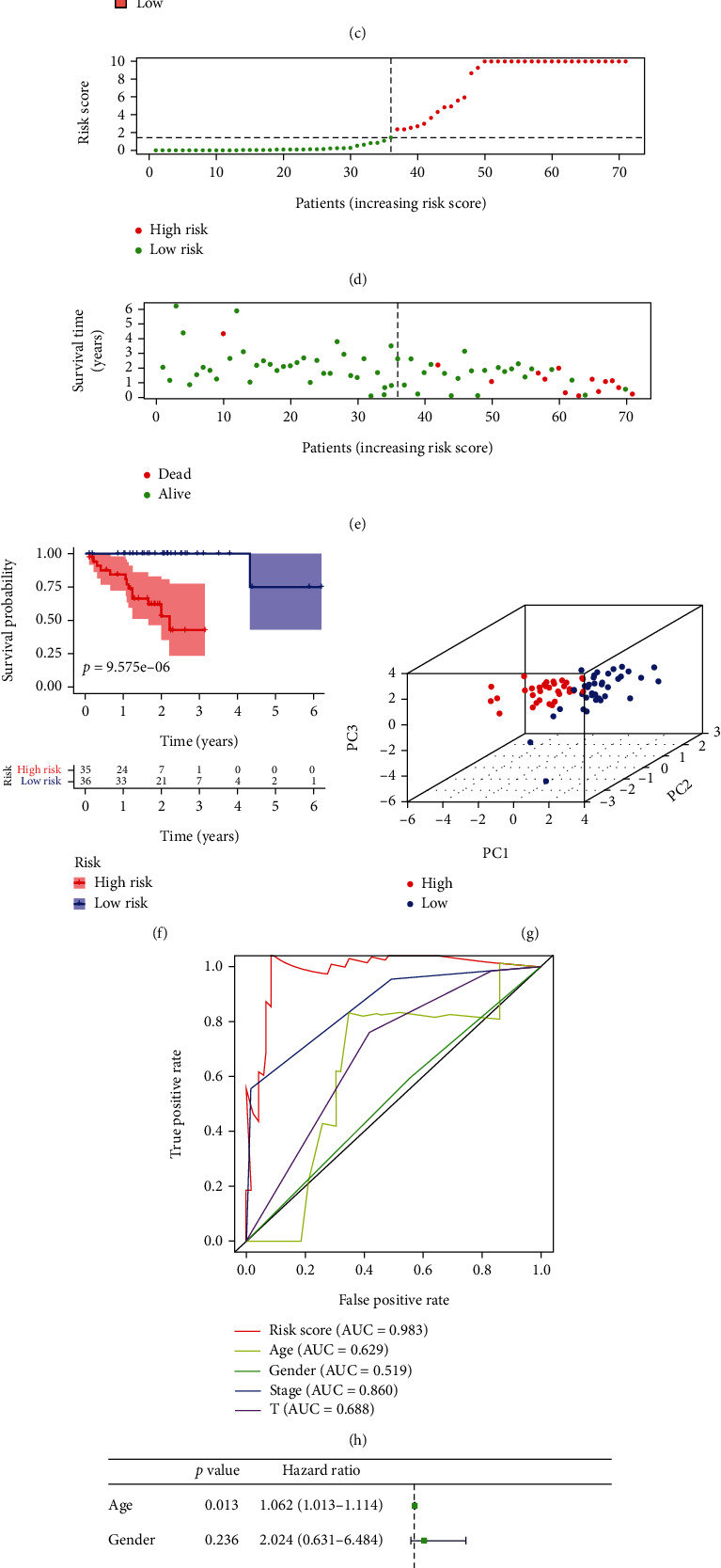
Prognostic value of autophagy-related lncRNAs in UM patients. (a) Flow diagram of the main process of the work. (b) Forest plot of the prognostic signature for predicting UM patient OS by univariate Cox regression analysis. (c) The expression heatmap of the 7 autophagy-related lncRNAs. (d) The risk score of each patient. (e) The survival status of each patient. (f) OS curves for UM patients in the high-risk group and the low-risk group. (g) PCA of the lncRNA signature in the high-risk and low-risk groups. (h) The receiver operating characteristic (ROC) curve of the risk score and clinical parameters in predicting the 5-year survival rate of UM patients. (i, j) The clinical parameters in the univariate and multivariate Cox regression analyses.

We downloaded and screened autophagy-related lncRNA expression profiles and patient clinical information from the TCGA database and performed prognostic analyses (univariate Cox, multivariate Cox, and survival analyses). Then, the seven top lncRNAs (AP003325.1, LINC01637, AC036214.2, UBXN10-AS1, AC090617.5, LINC01278, and SOX1-OT) associated with autophagy were screened according to risk scores, and prognostic, clinical, and experimental verification analyses were performed to elucidate their importance in UM (Figure 1(a)). Univariate analysis identified lncRNAs with significant positive (CYTOR, LHFPL3-AS1, AP000254.1, AP003352.1, UBR5-AS1, AC021087.3, LINC01637, HRAT92, PVT1, AC104129.1, AC009902.2, AL354836.1, AC109322.1, SOX1-OT, LINC01569, AL162457.2, AC036214.2, and AL445524.1) and negative (A1BG-AS1, AC069281.2, LINC01006, SNHG18, UBXN10-AS1, AC090617.5, and LINC01278) associations with poor survival in UM patients (Figure 1(b)). We obtained the risk scores of patients with UM with the following formula: risk score = (0.457476957109815 × expression of AP003352.1) + (0.277932228643516 × expression of LINC01637) + (−1.74709929211298 × expression of UBXN10 − AS1) + (−1.25063212596866 × expression of AC090617.5) + (−0.492626286784675 × expression of LINC01278) + (0.0848176378995068 × expression of SOX1 − OT) + (−0.606663063291623 × expression of AC036214.2). All patients with UM were divided into high-risk and low-risk groups based on the median value of the risk score. Figures 1(c)–1(e) shows the expression heatmap for the 7 autophagy-related lncRNAs (Figure 1(c)), the risk score of each patient (Figure 1(f)), and the survival status of each patient (Figure 1(g)). As shown in Figure 1(f), the high-risk UM melanoma patients had shorter survival (*p* < 0.001) than the low-risk group. As shown in Figure S1, 7 lncRNAs were associated with ARGs and divided into two groups for the risk assessment model. Moreover, the 3D principal component analysis (PCA) results showed that there was no significant overlap between the low-risk group and the high-risk group, as they were clustered separately, indicating that the risk scoring model based on the seven lncRNAs is feasible for UM patients (Figure 1(g)). The area under the curve (AUC) for the risk score was 0.983, indicating it was a better predictor of 5-year survival than other factors (age, tumour grade, pathological stage, and T stage) in UM patients (Figure 1(h)). Univariate analysis showed that age (HR, 1.062; 95%, 1.013–1.114; *p* = 0.013), pathological stage (HR, 6.160; 95%, 1.731–21.919; *p* = 0.005), and risk score (HR, 1.002; 95%, 1.001–1.003; *p* < 0.001) were significant predictors of survival (Figure 1(i)). Multivariate analysis showed that increasing age (HR, 1.059; 95%, 1.003-1.118; *p* = 0.038) was a significant independent predictor of poor overall survival (OS) (Figure 1(j)).

### 3.2. Clinical and Prognostic Analysis of LINC01278 in UM

**Figure 2:**
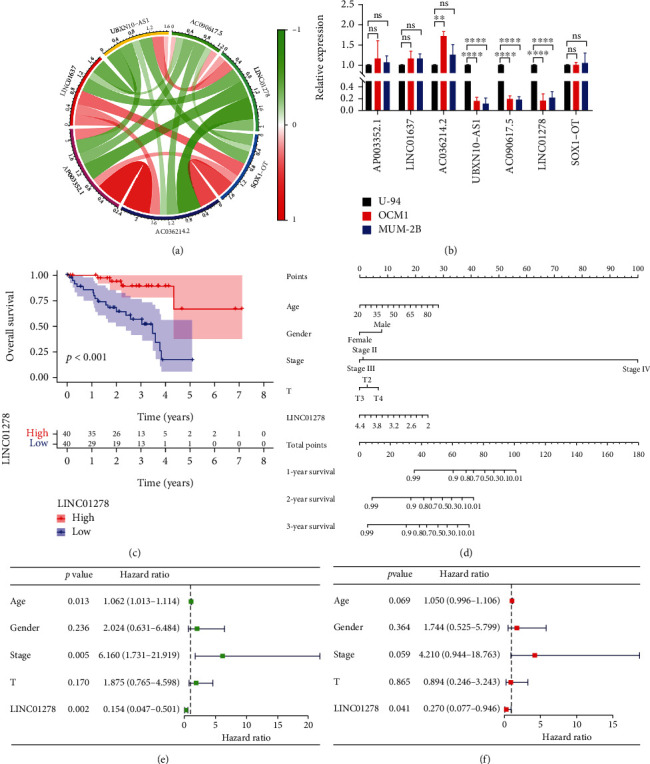
Prognostic value of LINC01278 in UM patients. (a) Correlation circle graph of 7 autophagy-related lncRNAs in UM. (b) The expression of 7 autophagy-related lncRNA mRNAs in U-94 and UM cells. (c) OS curves for UM patients in the LINC01278 high-expression group and the LINC01278 low-expression group. (d) The nomogram integrating LINC01278 score, age, sex, stage, and T predicted the probability of 1-, 2-, and 3-year OS. (e, f) The clinical parameters in the univariate and multivariate Cox regression analyses. (Data are presented as the mean ± SD; *n* = 3; ns: no significant difference, ^∗^*p* < 0.05, ^∗∗^*p* < 0.01, ^∗∗∗^*p* < 0.001, ^∗∗∗∗^*p* < 0.0001).

Then, we used the correlation circle chart to analyse the correlations between the above seven lncRNAs (Figure 2(a)). As shown in Figure 2(a), LINC01278 was highly correlated with the 7 autophagy-related lncRNAs. The expression of 7 autophagy-related lncRNAs in uveal melanocytes (U-94) and UM cells (OCM1 and MUM-2B) was detected by RT–qPCR, and only UBXN10-AS1, AC090617.5, and LINC01278 were significantly downregulated in UM cells compared with U-94 cells (Figure 2(b)). In addition, we analysed the risk types of the 7 lncRNAs mentioned above and their correlation with survival status. These results suggest that LINC01278 might play an important role in the progression of UM.

Based on the TCGA database (Table S1), we analysed the relationship between the expression of LINC01278 and clinical indicators. The expression of LINC01278 in stage IV patients was significantly higher than that in stage II patients (*p* < 0.001) and stage III patients (*p* = 0.0079) (Figure S2B). LINC01278 expression was significantly higher in patients with distant metastasis than in patients without metastasis (*p* = 0.0056) (Figure S2D). LINC01278 expression showed no significant difference in groups sorted by age, T stage, or sex (Figure S2A, C, and E). Kaplan-Meier's survival curves were used to analyse the relationship between LINC01278 expression and the OS rate, and the results suggested that low LINC01278 expression was significantly correlated with poor OS (*p* < 0.001) (Figure 2(c)). We predicted the 1-year, 2-year, and 3-year survival rates of UM patients by using clinical indicators (age, sex, pathological stage, and T stage) and LINC01278 expression in the nomogram model (Figure 2(d)). Univariate analysis showed that age (HR, 1.062; 95%, 1.013–1.114; *p* = 0.013), pathological stage (HR, 6.160; 95%, 1.731–21.919; *p* = 0.005), and LINC01278 expression (HR, 0.154; 95%, 0.047–0.501; *p* = 0.002) were significant predictors of survival (Figure 2(e)). Multivariate analysis showed that decreased LINC01278 expression (HR, 0.270; 95%, 0.077–0.946; *p* = 0.041) was a significant independent predictor of poor OS (Figure 2(f)).

### 3.3. LINC01278 Inhibits Malignant Phenotypes of UM Cells In Vitro and In Vivo

**Figure 3:**
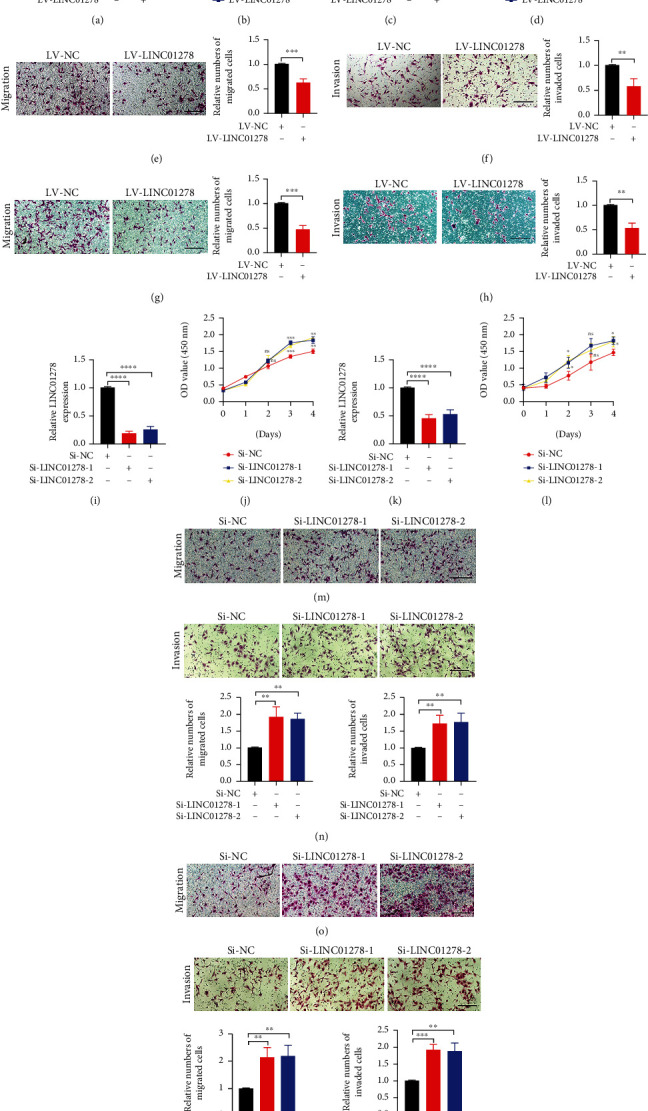
LINC01278 inhibits malignant phenotypes of UM cells in vitro. (a, c) The expression of LINC01278 mRNA in UM cells overexpressing LINC01278. (b, d) Analysis of the proliferation of UM cells overexpressing LINC01278. (e, g) Analysis of the migration of UM cells overexpressing LINC01278. (f, h) Analysis of the invasion of UM cells overexpressing LINC01278. (i, k) The expression of LINC01278 mRNA in UM cells with LINC01278 knockdown. (j, l) Analysis of the proliferation of UM cells with LINC01278 knockdown. (m, o) Analysis of the migration of UM cells with LINC01278 knockdown. (n, p) Analysis of the invasion of UM cells with LINC01278 knockdown. (OCM1 cells: (a, b), (e, f), (i, j), (m, n); MUM-2B cells: (c, d), (g, h), (k, l), (o, p); scale bar: 100 *μ*m; data are presented as the mean ± SD; *n* = 3; ^∗^*p* < 0.05, ^∗∗^*p* < 0.01, ^∗∗∗^*p* < 0.001, ^∗∗∗∗^*p* < 0.0001).

**Figure 4:**
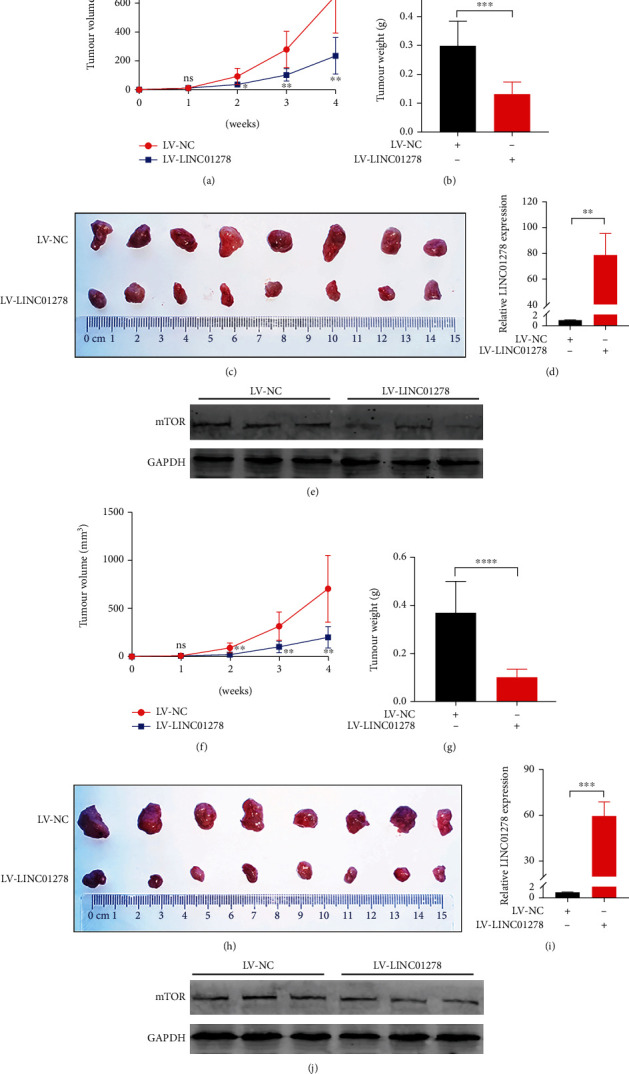
LINC01278 inhibits UM growth in vivo. (a, f) The volume of tumours formed in the LV-NC group and LV-LINC01278 group. (b, g) The weight of tumours formed in the LV-NC group and LV-LINC01278 group. (c, h) Images of tumours from the LV-NC group and LV-LINC01278 group. (d, i) The expression of LINC01278 mRNA in the LV-NC group and LV-LINC01278 group. (e, j) Western blot analysis of mTOR in the LV-NC group and LV-LINC01278 group. (OCM1 cells: (a–e); MUM-2B cells: (f–j); data are presented as the mean ± SD; *n* = 3 − 8; ^∗^*p* < 0.05, ^∗∗^*p* < 0.01, ^∗∗∗^*p* < 0.001,).

The expression of LINC01278 in the cytoplasm and nucleus was analysed by RT–qPCR, and the data showed that more than 75% of LINC01278 was located in the cytoplasm of UM cells (Figure S3A-B). To investigate the role of LINC01278 in UM, we overexpressed LINC01278 in two UM cell lines (OCM1 and MUM-2B) (Figures 3(a) and 3(c)). The results showed that the expression of LINC01278 was increased by more than 80-fold (OCM1) and 60-fold (MUM-2B) in the LV-LINC01278 group compared with the LV-NC group. Subsequently, we analysed the malignant phenotypes of UM cells, and the proliferation, migration, and invasion levels of the LV-LINC01278 groups of OCM1 and MUM-2B cells were lower than those of the LV-NC groups (Figures 3(b) and 3(d), S4A-B, S4E-F, and 3E-H). Similarly, we knocked down the expression of LINC01278 (Figures 3(i) and 3(k)) and analysed the growth of UM cells, and the results showed that the proliferation, migration, and invasion levels of the Si-LINC01278 groups were higher than those of the Si-NC group (Figures 3(j), 3(l), and 3(m)–3(p), S4C-D and S4G-H). Finally, LV-LINC01278 cell lines and LV-NC cell lines were subcutaneously injected into nude mice, and we examined the expression of LINC01278 mRNA in the two groups of tumours (Figures 4(d) and 4(i)); the tumours were photographed 4 weeks later, and the appearance, volume, and weight were analysed. We found that the volume and weight of tumours in the LV-LINC01278 group were significantly lower than those in the LV-NC group (Figures 4(a)–4(c) and 4(f)–4(h)). Also, we detected apoptosis and cell cycle regulator expression in knockdown or overexpression LINC01278 cell lines. As shown in Figure S5 and S6, LINC01278 promotes apoptosis and inhibits cell cycle progression in UM cells. In conclusion, LINC01278 can inhibit UM tumorigenesis in vitro and in vivo.

### 3.4. LINC01278 Suppresses the Growth of UM Cells by Promoting Autophagy

**Figure 5:**
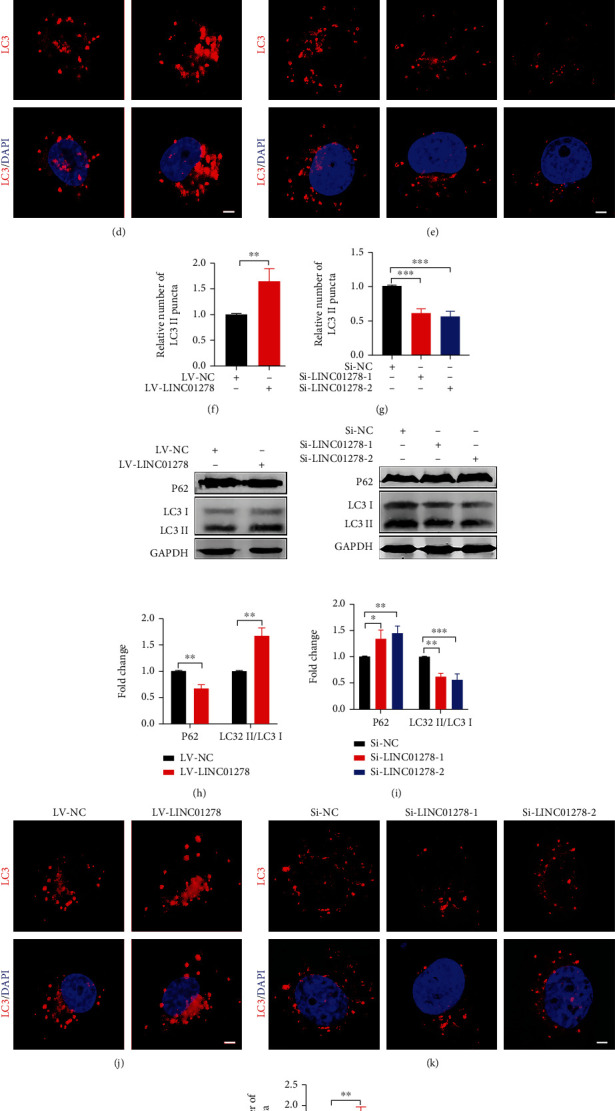
LINC01278 induces autophagy in UM. (a) GSEA of LINC01278 in UM patients. (b, h) Western blot analysis of LC3 and P62 in UM cells overexpressing LINC01278. (c, i) Western blot analysis of LC3 and P62 in UM cells with LINC01278 knockdown. (d, j) Immunofluorescence analysis of autophagosomes in UM cells overexpressing LINC01278. (e, k) Immunofluorescence analysis of autophagosomes in UM cells with LINC01278 knockdown. (f, g) Quantification of autophagosomes based on the results of (d) and (e). (l, m) Quantification of autophagosomes based on the results of (j) and (k). (OCM1 cells: (b–g); MUM-2B cells: (h–m); scale bar: 20 *μ*m; data are presented as the mean ± SD; *n* = 3; ^∗^*p* < 0.05, ^∗∗^*p* < 0.01, ^∗∗∗^*p* < 0.001).

Gene set enrichment analysis (GSEA) based on the TCGA database suggested that LINC01278 is related to the autophagy regulatory pathway (Figure 5(a)). In OCM1 and MUM-2B cells, compared with that in the LV-NC group, the LC3 II/LC3 I ratio in the LV-LINC01278 group was significantly increased, while P62 expression was significantly decreased (Figures 5(b) and 5(h)). According to immunofluorescence analysis, the number of autophagosomes in the LV-LINC01278 group was higher than that in the LV-NC group (Figures 5(d), 5(f), 5(j), and 5(l)). The above results indicated that overexpression of LINC01278 in UM cells promoted the autophagy pathway. Likewise, the ratio of LC3 II/LC3 I was significantly decreased, and P62 expression was significantly upregulated in the Si-LINC01278 groups compared with the Si-NC groups (Figures 5(c) and 5(i)). According to the immunofluorescence results, there were significantly fewer autophagosomes in the Si-LINC01278 groups than in the Si-NC groups (Figures 5(e), 5(g), 5(k), and 5(m)). This finding suggested that knockdown of LINC01278 in UM cells inhibits the autophagy pathway.

**Figure 6:**
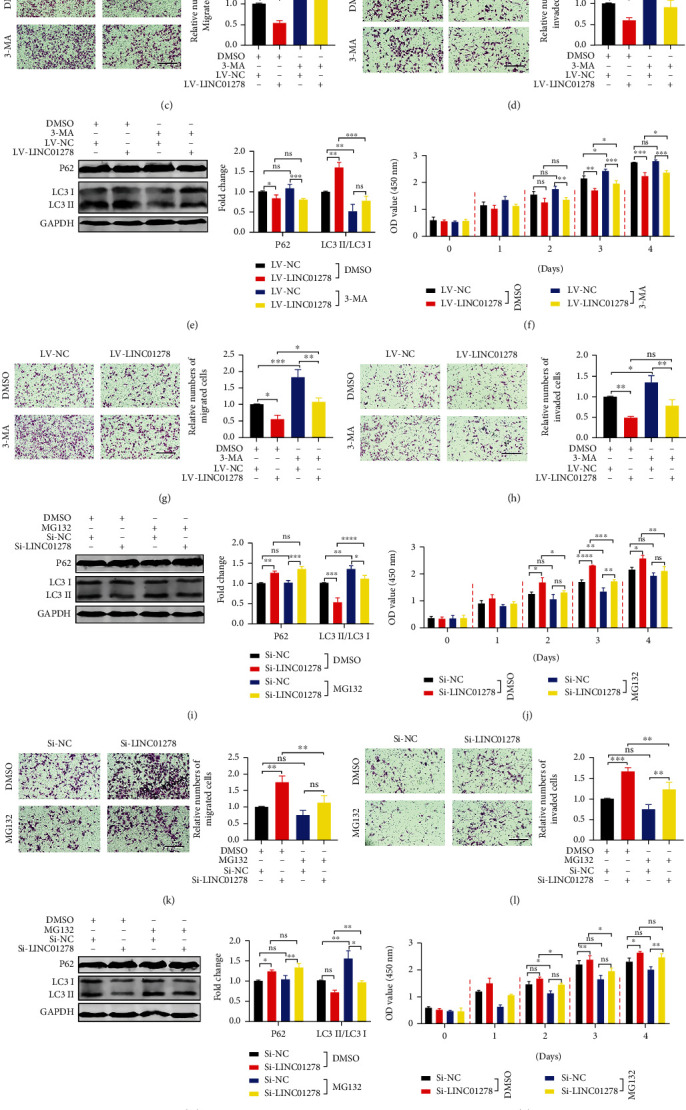
LINC01278 suppresses the growth of UM cells by promoting autophagy. (a, e) Western blot analysis of LC3 and P62 in the LV-NC group and LV-LINC01278 group after treatment with DMSO or 3-MA. (b, f) Proliferation analyses of the LV-NC group and LV-LINC01278 group after treatment with DMSO or 3-MA. (c, g) Migration analyses of the LV-NC group and LV-LINC01278 group after treatment with DMSO or 3-MA. (d, h) Invasion analyses of the LV-NC group and LV-LINC01278 group after treatment with DMSO or 3-MA. (i, m) Western blot analysis of LC3 and P62 in the Si-NC group and Si-LINC01278 group after treatment with DMSO or MG-132. (j, n) Proliferation analyses of the Si-NC group and Si-LINC01278 group after treatment with DMSO or MG-132. (k, o) Migration analyses of the Si-NC group and Si-LINC01278 group after treatment with DMSO or MG-132. (l, p) Invasion analyses of the Si-NC group and Si-LINC01278 group after treatment with DMSO or MG-132. (OCM1 cells: (a–d), (i–l); MUM-2B cells: (e–h); (m–p); scale bar: 100 *μ*m; data are presented as the mean ± SD; *n* = 3; ns: no significant difference, ^∗^*p* < 0.05, ^∗∗^*p* < 0.01, ^∗∗∗^*p* < 0.001, ^∗∗∗∗^*p* < 0.0001).

Next, we aimed to determine whether LINC01278 affects the growth of UM cells through the autophagy pathway. We investigated the optimal concentrations of an autophagy inhibitor (3-MA) and an autophagy agonist (MG-132) in UM cells (OCM1 and MUM-2B), and the data showed that the optimal concentrations were 30 *μ*mol/L and 40 *μ*mol/L, respectively (Figure S7A-D). We detected autophagy-related protein expression, proliferation, migration, and invasion after adding 3-MA or DMSO to UM cells in the LV-NC group and LV-LINC01278 group. First, regardless of DMSO and 3-MA treatment, the LC3 II/LC3 I ratio in the LV-LINC01278 group was significantly increased compared with that in the LV-NC group, while P62 expression and UM proliferation, migration, and invasion were decreased (Figures 6(a)–6(h), S8A, S8C, S8E, and S8G). Compared with that in the DMSO treatment group, the LC3 II/LC3 I ratio of UM cells (LV-NC and LV-LINC01278) after 3-MA treatment was significantly downregulated, while P62 expression and UM proliferation, migration, and invasion were significantly increased (Figures 6(a)–6(h), S8A, S8C, S8E, and S8G). Similarly, regardless of the addition of MG-132 or DMSO, the LC3 II/LC3 I ratio of the Si-LINC01278 group was significantly decreased compared with that of the Si-NC group, and P62 expression and the levels of UM proliferation, migration, and invasion were increased (Figures 6(i)–6(p), S8B, S8D, S8F, and S8H). Compared with that in the DMSO treatment group, the LC3 II/LC3 I ratio of UM cells (Si-NC and Si-LINC01278) treated with MG-132 was significantly increased, while P62 expression and the levels of UM proliferation, migration, and invasion were decreased (Figures 6(i)–6(p), S8B, S8D, S8F, and S8H). In conclusion, LINC01278 can suppress the growth of UM cells by promoting autophagy.

### 3.5. LINC01278 Inhibits UM Progression by Suppressing the mTOR Signalling Pathway

**Figure 7:**
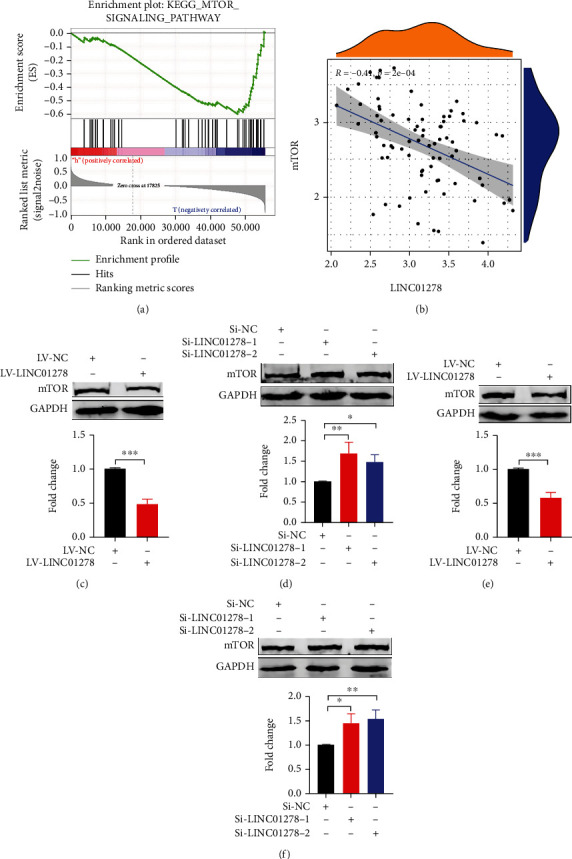
LINC01278 suppresses the mTOR signalling pathway. (a) Enrichment of the mTOR signalling pathway in the low LINC01278 expression group via GSEA. (b) The correlation of mTOR and LINC01278 expression in UM patients. (c, e) Western blot analysis of mTOR in UM cells overexpressing LINC01278. (d, f) Western blot analysis of mTOR in UM cells with LINC01278 knockdown. (OCM1 cells: (c, d); MUM-2B cells: (e, f); data are presented as the mean ± SD; *n* = 3; ^∗^*p* < 0.05, ^∗∗^*p* < 0.01, ^∗∗∗^*p* < 0.001).

The GSEA results of LINC01278 also showed that it was related to the mTOR signalling pathway (Figure 7(a)), and the correlation results showed that LINC01278 expression was negatively correlated with mTOR expression (*R* = −0.41, *p* < 0.001) (Figure 7(b)). We examined mTOR protein expression after LINC01278 was overexpressed or knocked down in UM cells. Compared with that in the LV-NC group, mTOR protein expression was downregulated in the LV-LINC01278 group (Figures 7(c) and 7(e)). Compared with that in the Si-NC groups, mTOR protein expression was upregulated in the Si-LINC01278 groups (Figures 7(d) and 7(f)). Also, we examined the expression of mTOR protein in the LV-NC and LV-LINC01278 groups in vivo (Figures 4(e) and 4(j)). These data suggested that LINC01278 inhibits the protein expression of mTOR.

**Figure 8:**
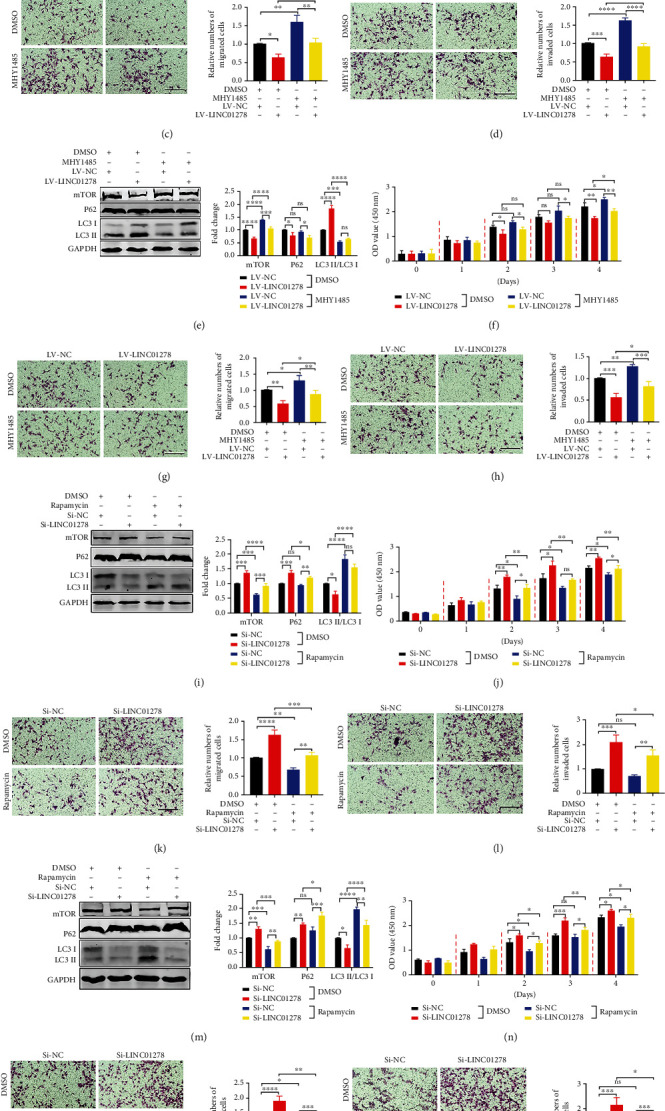
LINC01278 inhibits UM progression by suppressing the mTOR signalling pathway. (a, e) Western blot analysis of mTOR, LC3, and P62 in the LV-NC group and LV-LINC01278 group after treatment with DMSO or MHY1485. (b, f) Proliferation analyses of the LV-NC group and LV-LINC01278 group after treatment with DMSO or MHY1485. (c, g) Migration analyses of the LV-NC group and LV-LINC01278 group after treatment with DMSO or MHY1485. (d, h) Invasion analyses of the LV-NC group and LV-LINC01278 group after treatment with DMSO or MHY1485. (i, m) Western blot analysis of mTOR, LC3, and P62 in the Si-NC group and Si-LINC01278 group after treatment with DMSO or rapamycin. (j, n) Proliferation analyses of the Si-NC group and Si-LINC01278 group after treatment with DMSO or rapamycin. (k, o) Migration analyses of the Si-NC group and Si-LINC01278 group after treatment with DMSO or rapamycin. (l, p) Invasion analyses of the Si-NC group and Si-LINC01278 group after treatment with DMSO or rapamycin. (OCM1 cells: (a–d), (i–l); MUM-2B cells: (e–h), (m–p); scale bar: 100 *μ*m; data are presented as the mean ± SD; *n* = 3; ns: no significant difference, ^∗^*p* < 0.05, ^∗∗^*p* < 0.01, ^∗∗∗^*p* < 0.001, ^∗∗∗∗^*p* < 0.0001).

We further investigated whether LINC01278 affects the autophagy and UM progression through the mTOR pathway. First, we determined the optimal concentrations of an mTOR agonist (MHY1485, 40 *μ*mol/L) and mTOR inhibitor (rapamycin, 30 *μ*mol/L) in UM cells (OCM1 and MUM-2B) (Figure S9A-D). We detected mTOR protein expression, autophagy-associated protein expression, and malignant phenotypes of UM cells after adding MHY1485 or DMSO to UM cells in the LV-NC group and LV-LINC01278 group. First, regardless of DMSO and MHY1485 treatment, the LC3 II/LC3 I ratio was increased in the LV-LINC01278 group compared with the LV-NC group, while mTOR expression, P62 expression, and the levels of UM proliferation, migration, and invasion were decreased (Figures 8(a)–8(h), S10A, S10C, S10E, and S10G). Compared with that in the DMSO treatment group, the LC3 II/LC3 I ratio of the UM cells treated with MHY1485 (LV-NC and LV-LINC01278) was significantly downregulated, while mTOR expression, P62 expression, and UM proliferation, migration, and invasion were increased (Figures 8(a)–8(h), S10A, S10C, S10E, and S10G). Similarly, regardless of the addition of rapamycin and DMSO, the LC3 II/LC3 I ratio was significantly decreased in the Si-LINC01278 group compared with the Si-NC group, and mTOR expression, P62 expression, and UM proliferation, migration, and invasion were increased (Figures 8(i)–8(p), S10B, S10D, S10F, and S10H). Compared with that in the DMSO treatment group, the LC3 II/LC3 I ratio of the rapamycin-treated UM cells (Si-NC and Si-LINC01278) was significantly increased, while mTOR expression, P62 expression, and the levels of UM proliferation, migration, and invasion were decreased (Figures 8(i)–8(p), S10B, S10D, S10F, and S10H). Finally, we overexpressed the mTOR plasmid in the LV-LINC01278 cell lines and then detected some malignant phenotypes of UM cells. As shown in Figure S11, overexpression of mTOR can inhibit the suppressive function of LINC01278 in UM cells. These data can suggest that LINC01278 inhibits the autophagy and growth of UM cells by suppressing mTOR.

**Figure 9:**
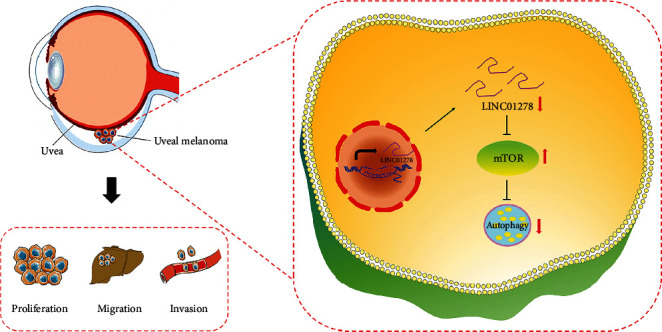
Schematic illustration of the LINC01278 mechanism in UM.

In summary, we concluded that the lncRNA LINC01278 induces autophagy to inhibit UM progression by suppressing the mTOR signalling pathway (Figure 9).

## 4. Discussion

In this study, we identified 7 autophagy-related lncRNAs closely associated with UM prognosis and developed a risk assessment model. We found that the model had good prognostic value in terms of predicting the 5-year survival of patients (AUC = 0.983) (Figure 1). Subsequently, we assessed the correlation of these lncRNAs with UM prognosis based on RT–qPCR mRNA expression data and previous literature reports, and a targeted lncRNA (LINC01278) that might play an important role in UM progression was selected (Figure 2). lncRNAs are involved in various diseases (cancer, ageing, neurodegeneration, and cardiovascular disease) and signalling pathways (p53, NF-*κ*B, PI3K/AKT and Notch) and play an important role in tumour proliferation, migration, and invasion as tumour-promoting molecules or tumour-inhibiting molecules [36–38]. lncRNAs are ideal targets for potential cancer treatments and have several characteristics: low concentration, fast turnover, specificity, multiple binding sites, and the ability to regulate chromatin modification and function [4]. Furthermore, lncRNAs play a role in UM tumorigenesis. For example, lncRNAs might affect the progression of UM by activating or inhibiting signalling pathways such as P53, mTOR, and AMPK [29, 39]. Oncogenic lncRNAs commonly promote angiogenesis; cell proliferation, migration, invasion, and apoptosis; and chemoresistance. However, antitumour lncRNAs have the opposite effects [40, 41]. These molecules might also serve as potential biomarkers and new diagnostic and therapeutic targets for UM.

We also analysed the correlation of LINC01278 expression with UM clinical characteristics and survival using the TCGA database. The results showed that decreased LINC01278 expression was associated with increased pathological stage and was also a significant independent predictor of poorer OS (Figure 2). The in vitro and in vivo experimental results further confirmed that high LINC01278 expression inhibited the proliferation, migration, and invasion of UM cells (Figure 3). We further explored the molecular mechanism by which LINC01278 inhibits UM progression. By analysing the GSEA results for LINC01278, we found that it is related to the autophagy pathway (Figure 5(a)). Therefore, we further explored whether LINC01278 inhibits the proliferation, migration, and invasion of UM cells through autophagy. We detected the expression of autophagy-related proteins and the number of autophagosomes after overexpressing or knocking down LINC01278. We found that the ratio of LC3 II/LC3 I and the number of autophagosomes increased with increasing LINC01278 expression, while the expression of P62 decreased. Moreover, the results were reversed when LINC01278 was knocked down (Figure 5). These results suggested that LINC01278 can induce the autophagy pathway in UM cells. Autophagy plays a dual role in tumour progression. In the early stage of the tumour, this process inhibits tumorigenesis by maintaining genomic stability and preventing cell damage and inflammation, while in the late tumour stage, it promotes tumour development by maintaining mitochondrial function and metabolism, reducing DNA damage, and enhancing cancer cell resistance [42–44]. We detected the autophagy-related protein expression, proliferation, migration, and invasion of UM cells treated with an autophagy inhibitor (3-MA) and an autophagy agonist (MG-132) (Figure 6). The results indicated that LINC01278 can inhibit the proliferation, migration, and invasion of UM cells by promoting autophagy.

mTOR constitutes the catalytic subunit of two distinct complexes (mTORC1 and mTORC2) and is closely related to cellular anabolism or catabolism, cell growth, autophagy, and various signalling pathways [45]. mTORC1, which is a multiprotein complex composed of mTOR, RAPTOR, MLST8 (or GPL), PRAS40, and DEPTOR, is involved in autophagy, protein synthesis, and the ribosome synthesis pathway. In particular, mTORC1 plays an important role in autophagy by regulating downstream targets (ULK1 complex, VSP34 complex, ATG5-RACK1 protein complex, DAP1, lysosomal biogenesis-related genes, and acetyltransferase P300). mTORC2, consisting of mTOR, RICTOR, SIN1, MLST8, and DEPTOR, is a bidirectional regulator of autophagy. On the one hand, the mTORC2/AKT1/FOXO3a signalling pathway inhibits autophagy. On the other hand, the mTORC2/IGF1 signalling pathway promotes autophagy [45–47]. The results of bioinformatic analysis showed that LINC01278 expression was negatively correlated with the mTOR signalling pathway in UM (Figure 7(a)). Furthermore, we verified that LINC01278 inhibits the expression of mTOR protein by overexpressing or knocking down LINC01278 in UM cells (Figure 7). We detected autophagy-related protein expression, proliferation, migration, and invasion in UM cells treated with an mTOR agonist (MHY1485) and an mTOR inhibitor (rapamycin) (Figure 8). We found that LINC01278 can induce autophagy to inhibit tumour progression by suppressing the mTOR signalling pathway.

This study has some limitations. Due to the lack of tissue samples from UM patients, the expression of LINC01278 was not validated in human tissues. Moreover, we still need to investigate whether LINC01278 can serve as a potential diagnostic marker in UM patients. Overall, LINC01278 induces autophagy to inhibit UM progression by suppressing the mTOR signalling pathway and may act as a new independent prognostic factor and potential therapeutic target gene.

## Data Availability

The data used to support the findings of this study are included within the article.

## Abbreviations

UM: Uveal melanoma
mTOR: Mechanistic target of rapamycin kinase
LC3: Microtubule associated protein 1 light chain 3 alpha
P62: Sequestosome 1
3-MA: 3-Methyladenine
ARGs: Autophagy-related genes
GSEA: Gene set enrichment analysis
ROC: Receiver operating characteristic curve
AUC: Area under the curve
OS: Overall survival
TCGA: The cancer genome atlas
GNAQ: G protein subunit alpha q
BECN1: Beclin 1
AXIIR: Annexin A2 receptor
AMPK: Protein kinase AMP-activated catalytic subunit alpha 1
Rab5: RAB5A, member RAS oncogene family
PTHR1: Parathyroid hormone 1 receptor
TCF-4: Transcription factor 4
Smad2/3: SMAD family member 2/3
DNM3: Dynamin 3
MAPK: Mitogen activated kinase-like protein
GNA11: G protein subunit alpha 11
PI3K: Phosphatidylinositol 3-kinase
MLST8: MTOR associated protein, LST8 homologue
DEPTOR: DEP domain containing MTOR interacting protein
ULK1: Unc-51 like autophagy activating kinase 1
VSP34: Putative atypical/PIKK/PI3K protein kinase
RICTOR: RAPTOR-independent companion of MTOR complex 2
AKT1: AKT serine/threonine kinase 1
IGF1: Insulin like growth factor 1.

## Supplementary Materials

Supplementary Materials Figure S1: Sankey's diagram of autophagy-related genes and related lncRNAs. Figure S2: association of LINC01278 expression with clinical parameters in UM patients. Figure S3: LINC01278 is mainly located in the cytoplasm of UM cells. Figure S4: LINC01278 inhibits the proliferation of UM cells in vitro. Figure S5: the effect of LINC01278 on UM apoptosis in vitro. Figure S6: the effect of LINC01278 on UM cell cycle in vitro. Figure S7: the optimum concentrations of MG-132 and 3-MA in UM cells. Figure S8: LINC01278 inhibits the proliferation of UM cells by inducing autophagy. Figure S9: the optimum concentrations of rapamycin and MHY1485 in UM cells. Figure S10: LINC01278 inhibits the proliferation of UM cells by suppressing the mTOR signalling pathway. Figure S11: LINC01278 inhibits UM progression by suppressing mTOR protein expression. Table S1: clinical characteristics of UM patients involved in the research.
