# Supplementary material for: LINC01278 Induces Autophagy to Inhibit Tumour Progression by Suppressing the mTOR Signalling Pathway
Source: Oxid Med Cell Longev. 2023 Jan 18;2023:8994901. doi: 10.1155/2023/8994901 (PMC9876672; doi:10.1155/2023/8994901)
Supplement: Supplementary Materials — Figure S1: Sankey's diagram of autophagy-related genes and related lncRNAs. Figure S2: association of LINC01278 expression with clinical parameters in UM patients. Figure S3: LINC01278 is mainly located in the cytoplasm of UM cells. Figure S4: LINC01278 inhibits the proliferation of UM cells in vitro. Figure S5: the effect of LINC01278 on UM apoptosis in vitro. Figure S6: the effect of LINC01278 on UM cell cycle in vitro. Figure S7: the optimum concentrations of MG-132 and 3-MA in UM cells. Figure S8: LINC01278 inhibits the proliferation of UM cells by inducing autophagy. Figure S9: the optimum concentrations of rapamycin and MHY1485 in UM cells. Figure S10: LINC01278 inhibits the proliferation of UM cells by suppressing the mTOR signalling pathway. Figure S11: LINC01278 inhibits UM progression by suppressing mTOR protein expression. Table S1: clinical characteristics of UM patients involved in the research. [file 8994901.f1.docx]

**Supplementary Materials:**


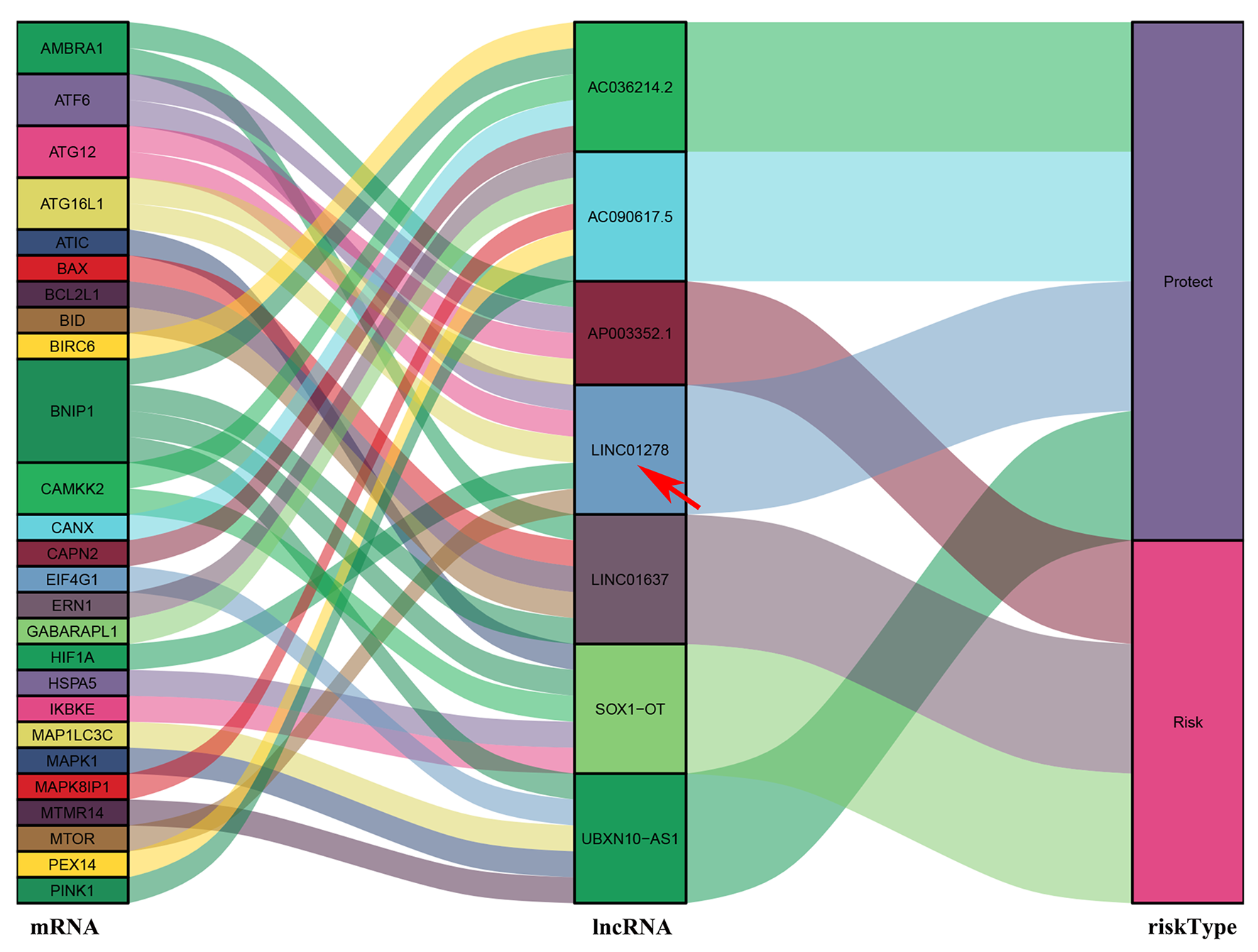


**Fig. S1 Sankey diagram of autophagy-related genes and related lncRNAs.**


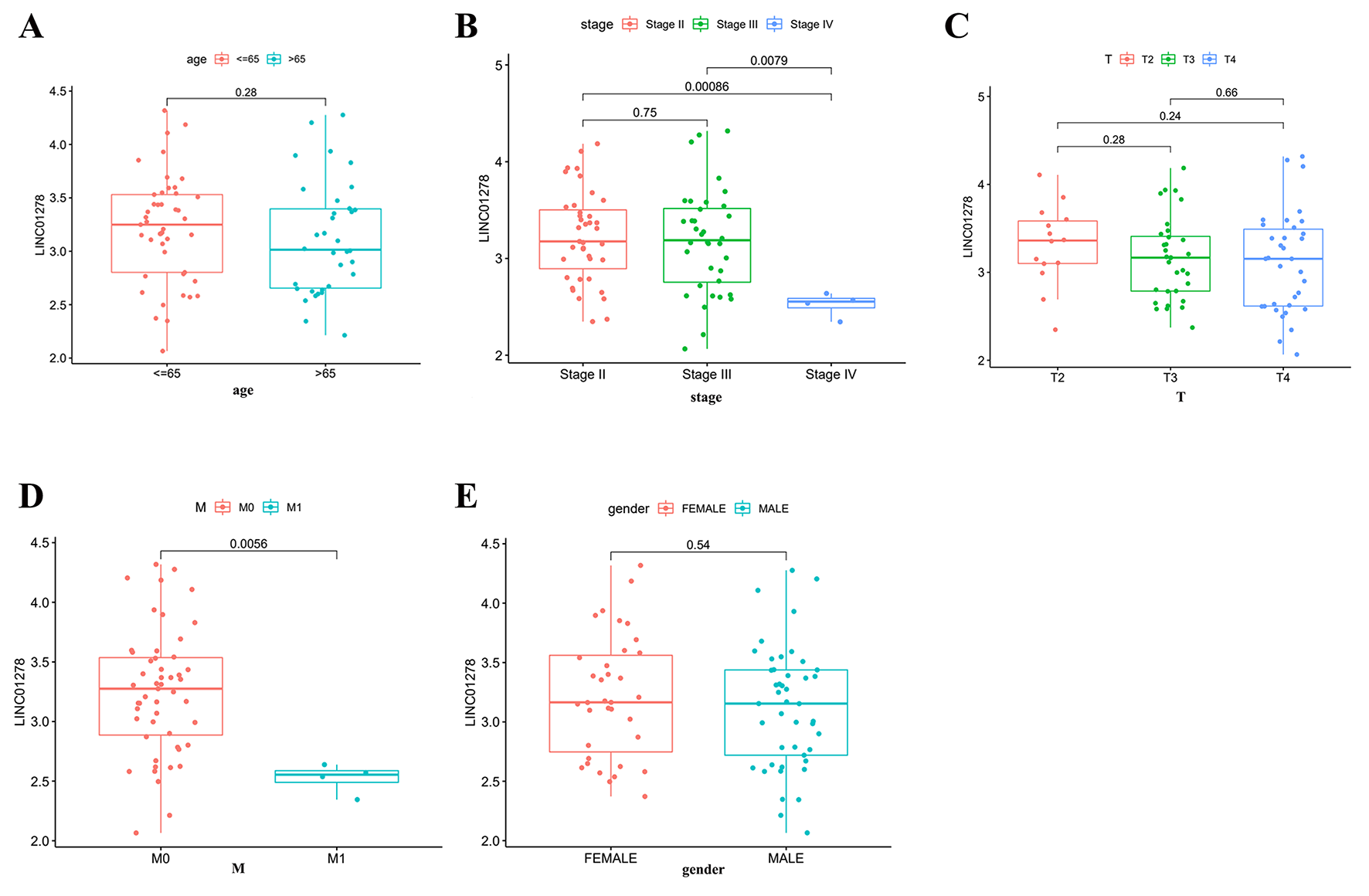


**Fig. S2 Association of LINC01278 expression with clinical parameters in UM patients.**

**(A)** Age (p > 0.05); **(B)** stage (p < 0.05); **(C)** T stage (p > 0.05); **(D)** M stage (p < 0.05); **(E)** sex (p > 0.05).


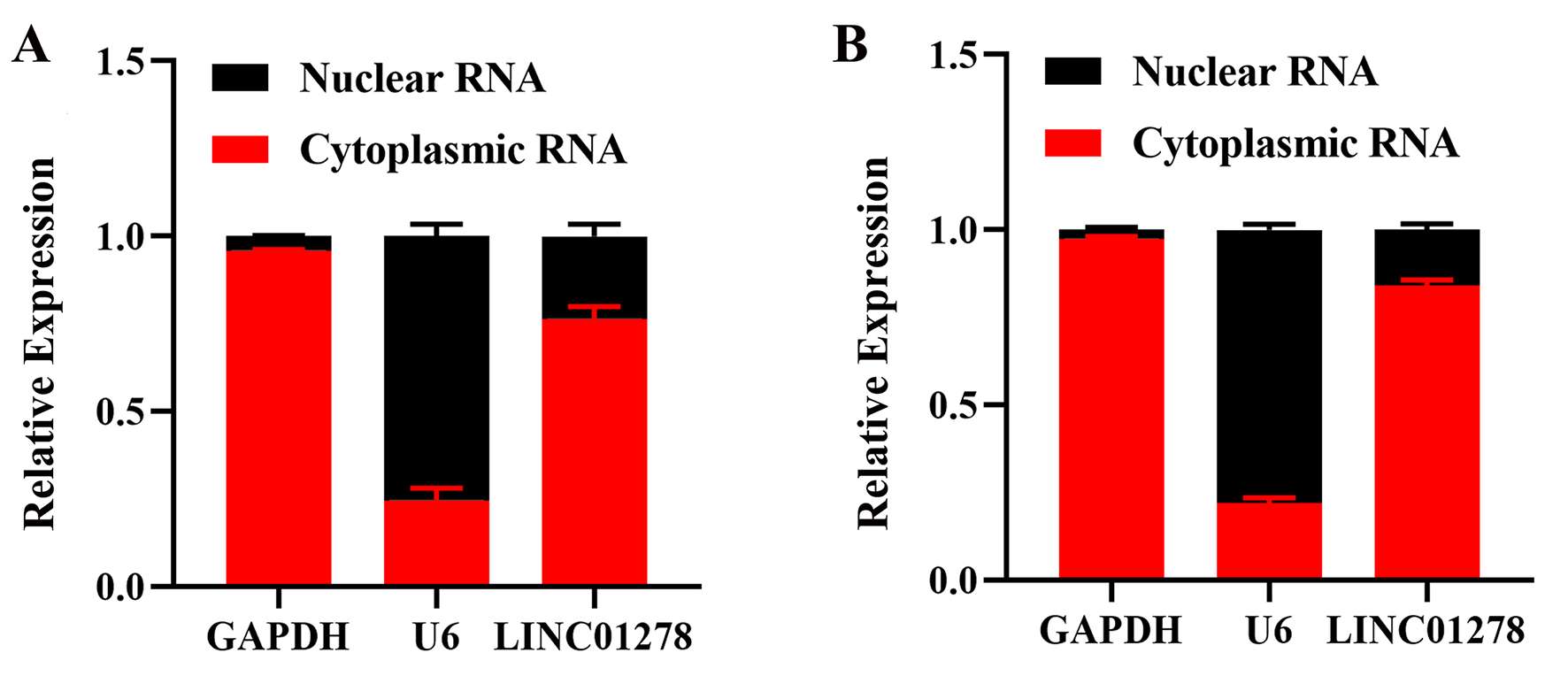


**Fig. S3 LINC01278 is mainly located in the cytoplasm of UM cells.**

**(A)** Analysis of the subcellular localization of LINC01278 in OCM1 cells. **(B)** Analysis of the subcellular localization of LINC01278 in MUM-2B cells.


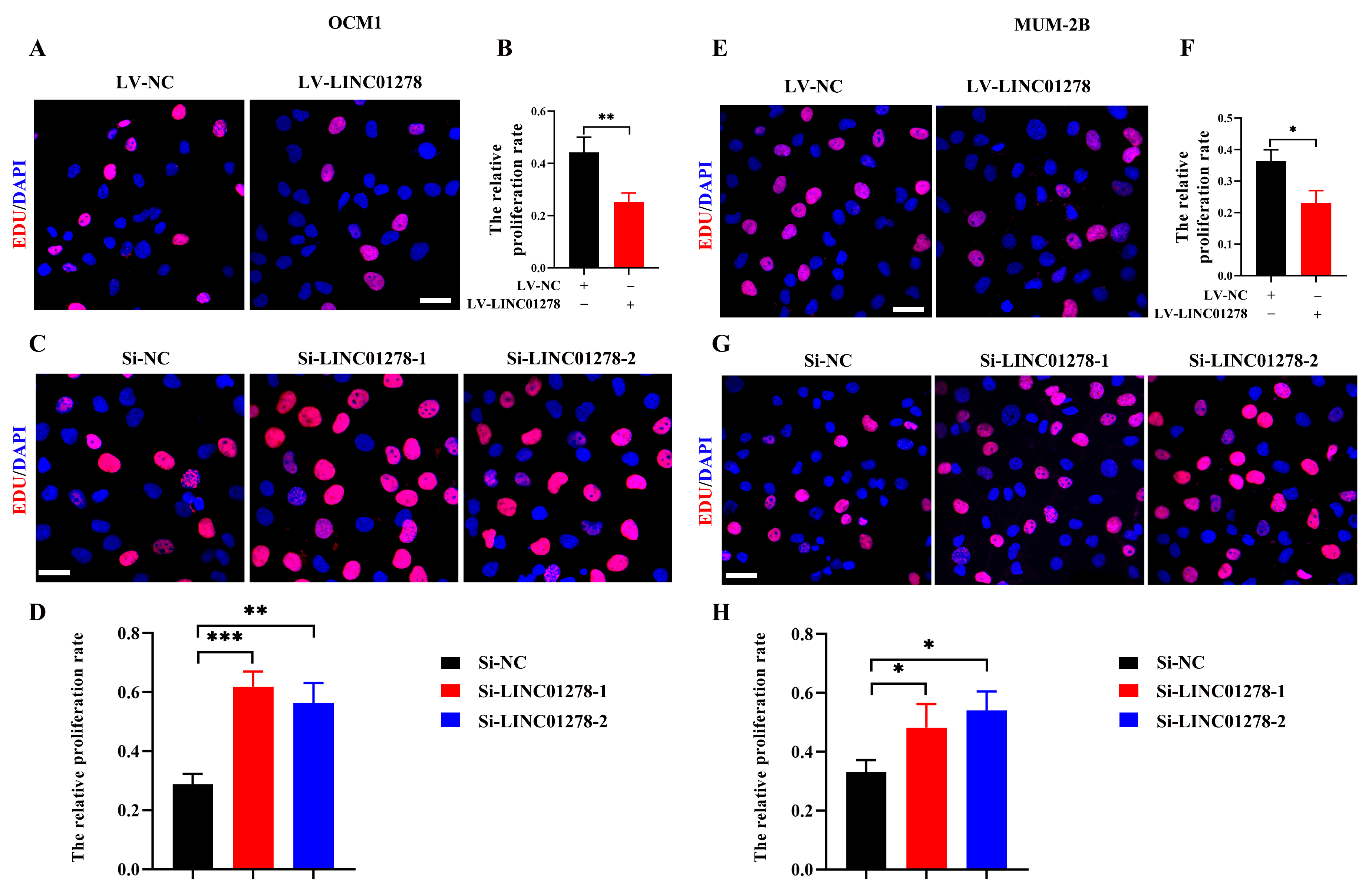


**Fig. S4 LINC01278 inhibits the proliferation of UM cells in vitro.**

**(A-B and E-F)** Analysis of the proliferation of UM cells overexpressing LINC01278 by the EDU assay. **(C-D and G-H)** Analysis of the proliferation of UM cells with LINC01278 knockdown by the EDU assay. (OCM1 cells: A-D; MUM-2B cells: E-H; scale bar: 60 µm; data are presented as the mean ± SD; n = 3; *p < 0.05, **p < 0.01, ***p < 0.001).

**
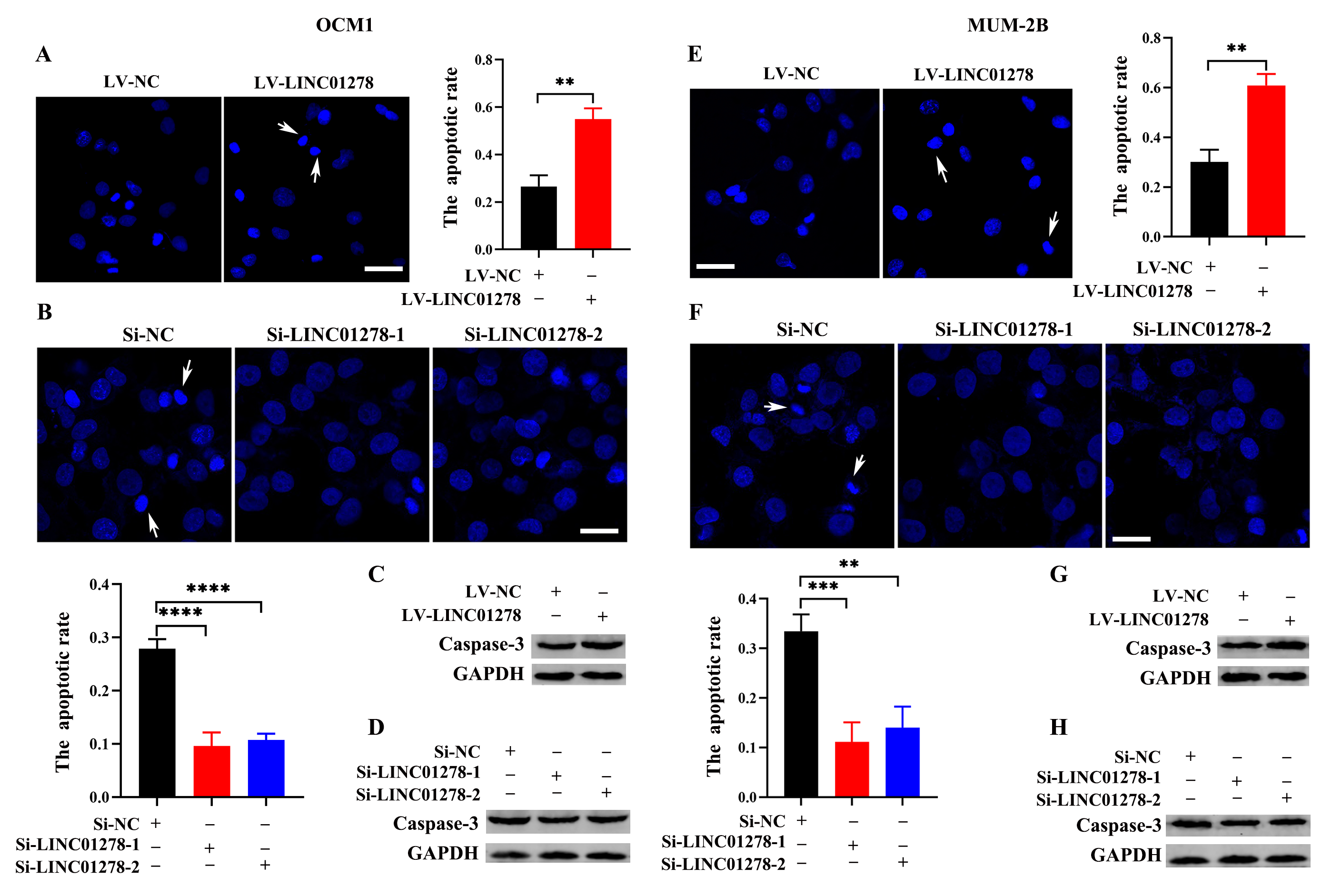
**

**Fig. S5** **The effect of LINC01278 on UM** **apoptosis in vitro.**

**(A and E)** Analysis of the apoptosis of UM cells overexpressing LINC01278. **(B and F)** Analysis of the apoptosis of UM cells with LINC01278 knockdown. **(C and G)** Western blot analysis of Caspase-3 in UM cells overexpressing LINC01278. **(D and H)** Western blot analysis of Caspase-3 in UM cells LINC01278 knockdown. (OCM1 cells: A-D; MUM-2B cells: E-H; scale bar: 60 µm; data are presented as the mean ± SD; n = 3; *p < 0.05, **p < 0.01, ***p < 0.001, **** p < 0.0001).

**
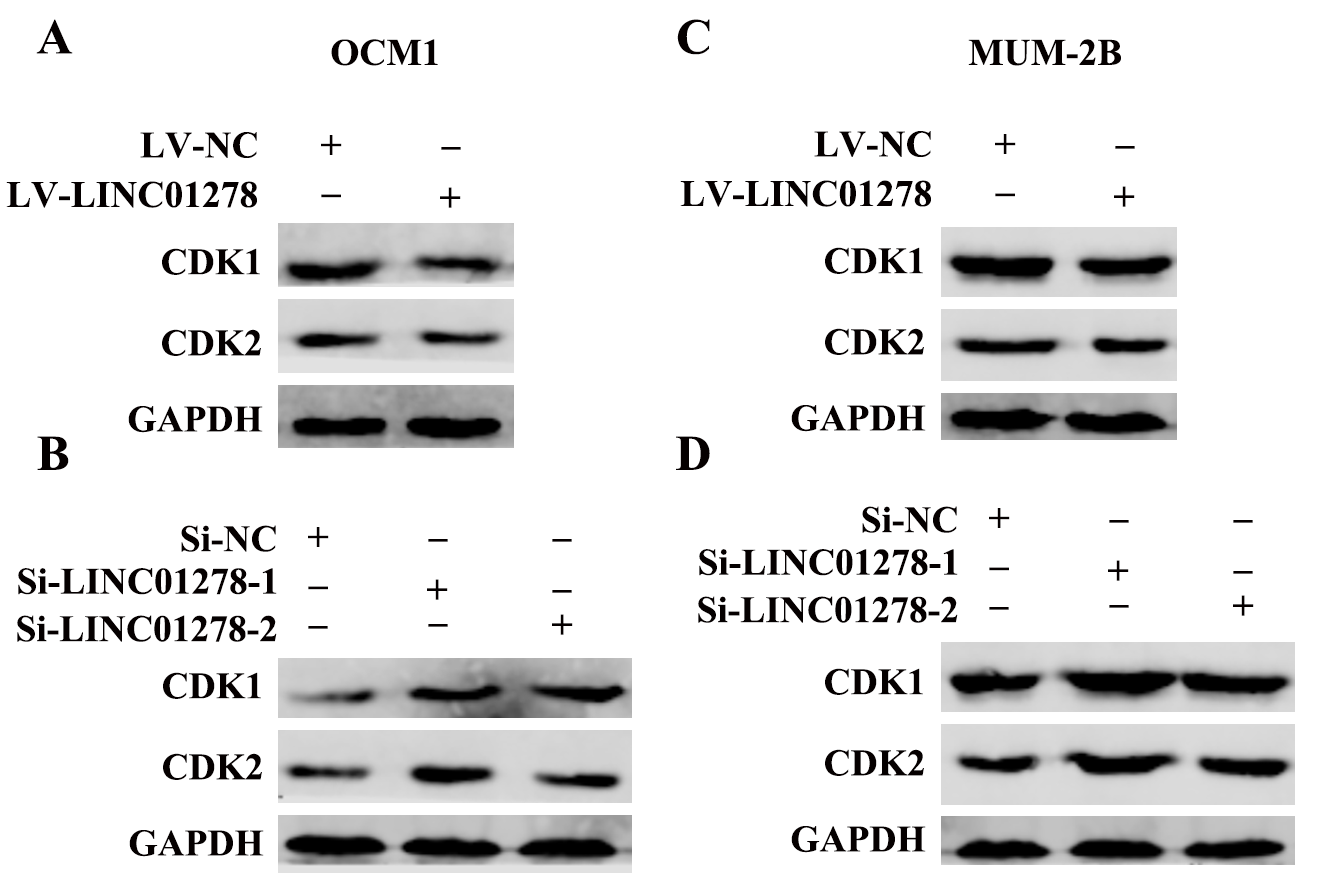
**

**Fig. S6 The effect of LINC01278 on UM cell cycle in vitro.**

**(A and C)** Western blot analysis of CDK1 and CDK2 in UM cells overexpressing LINC01278. **(B and D)** Western blot analysis of CDK1 and CDK2 in UM cells LINC01278 knockdown. (OCM1 cells: A-B; MUM-2B cells: C-D).

**
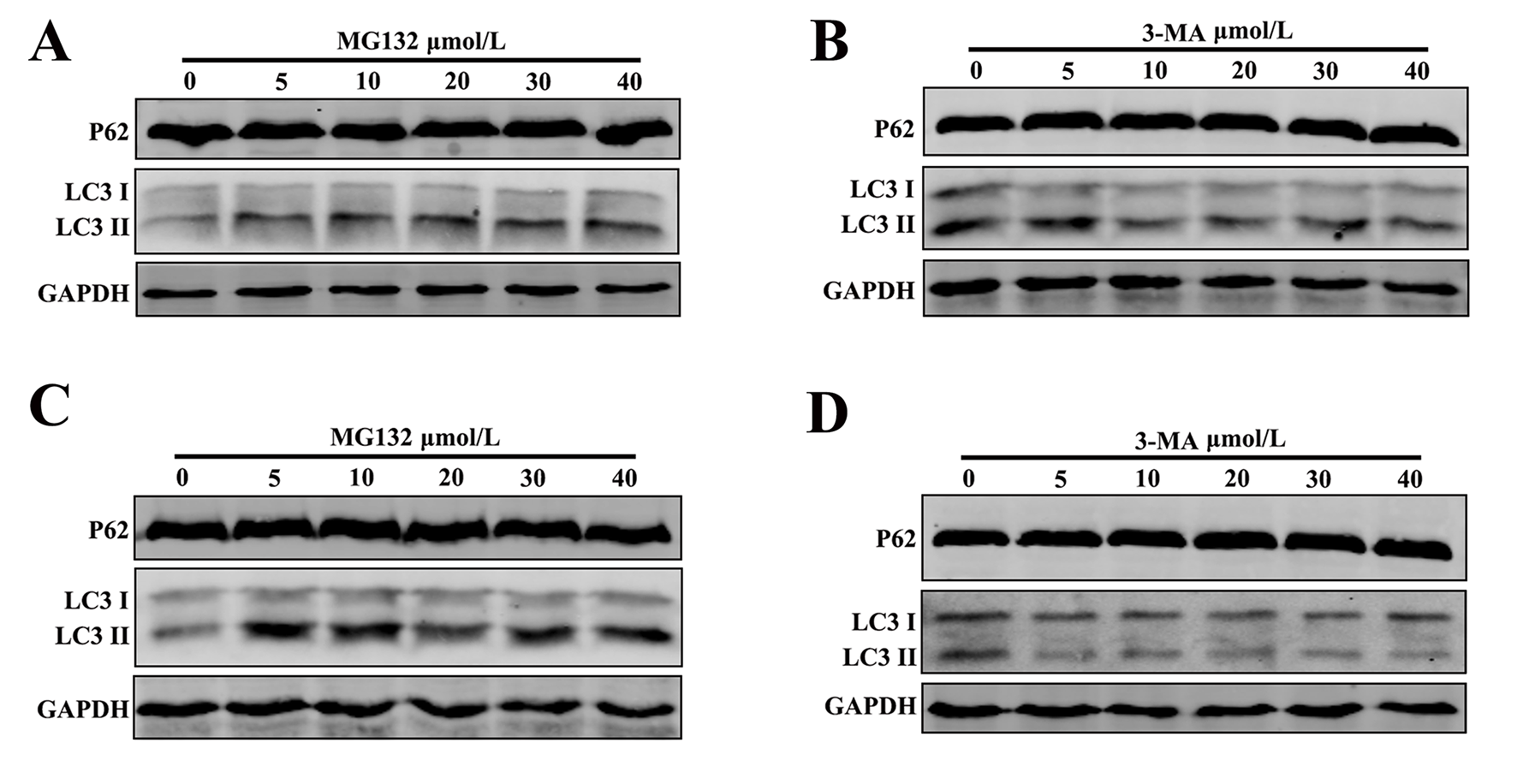
**

**Fig. S7 The optimum concentrations of MG-132 and 3-MA in UM cells.**

**(A and C)** Western blot analysis of LC3 and P62 in UM cells treated with different concentrations of MG-132. **(B and D)** Western blot analysis of LC3 and P62 in UM cells treated with different concentrations of 3-MA. (OCM1 cells: A-B; MUM-2B cells: C-D).


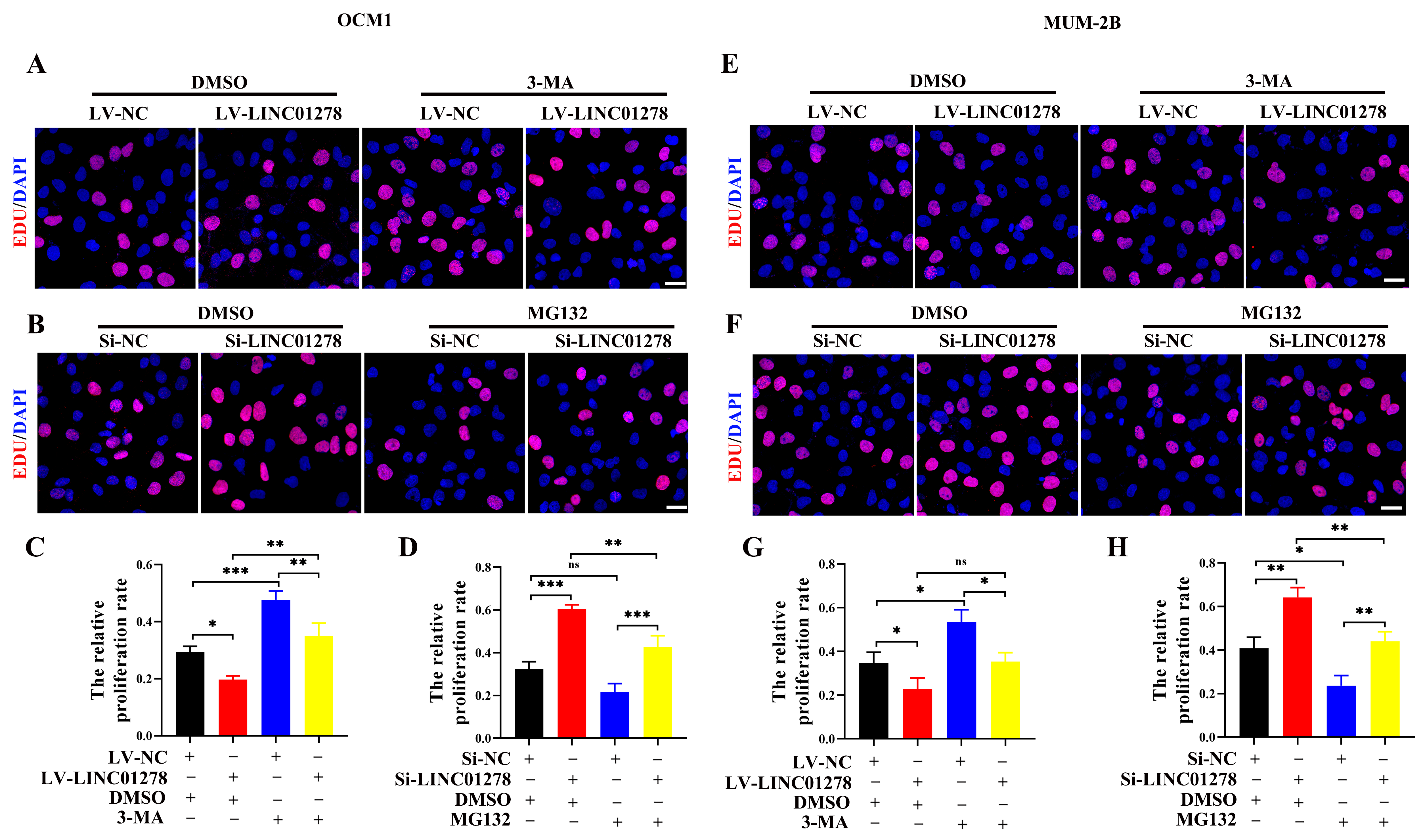


**Fig. S8** **LINC01278 inhibits the proliferation of UM cells by inducing autophagy**

**(A and E)** Proliferation analyses of the LV-NC group and LV-LINC01278 group after treatment with DMSO or 3-MA by the EDU assay. **(C and D)** C is the statistical results of A and D is the statistical results of B. **(B and F)** Proliferation analyses of the Si-NC group and Si-LINC01278 group after treatment with DMSO or MG-132 by the EDU assay. **(G and H)** G is the statistical results of E and H is the statistical results of F. (OCM1 cells: A-D; MUM-2B cells: E-H; scale bar: 60 µm; data are presented as the mean ± SD; n = 3; ns: no significant difference, *p < 0.05, **p < 0.01, ***p < 0.001).


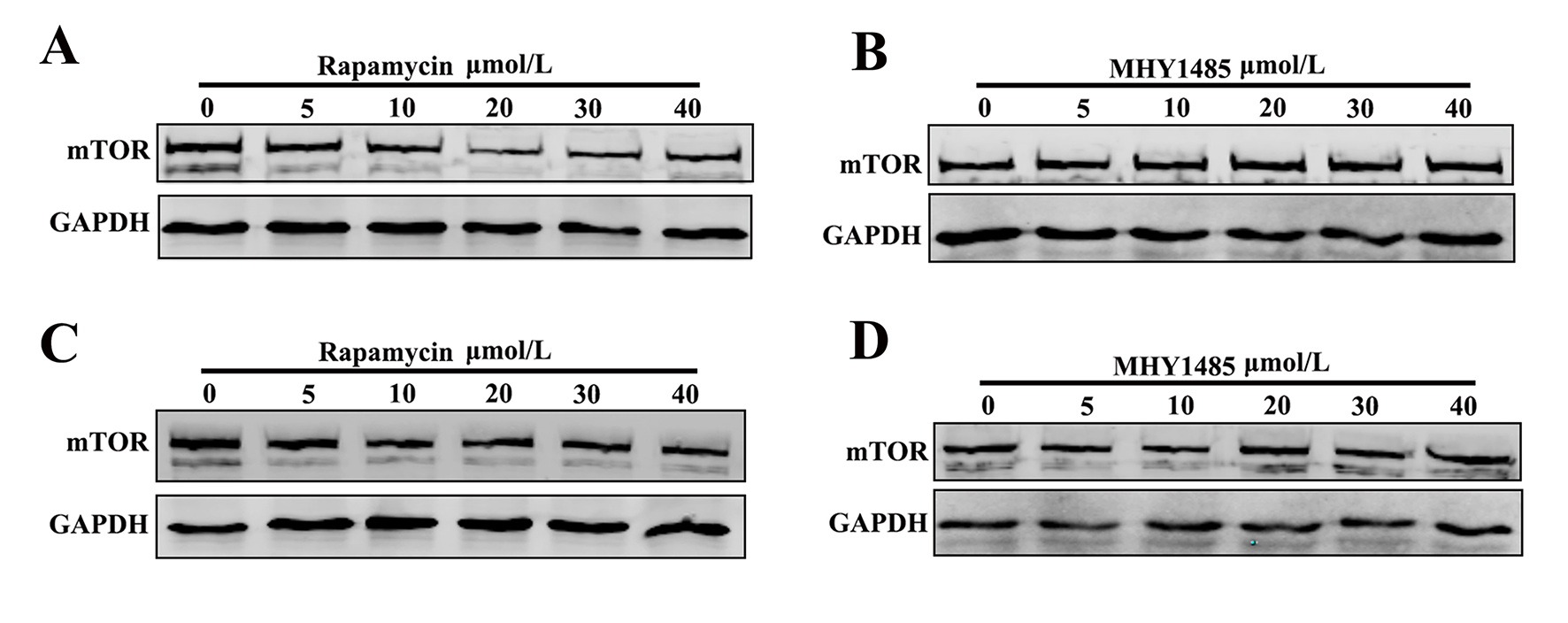
**Fig. S9 The optimum concentrations of rapamycin and MHY1485 in UM cells.**

**(A and C)** Western blot analysis of mTOR in UM cells treated with different concentrations of rapamycin. **(B and D)** Western blot analysis of mTOR in UM cells treated with different concentrations of MHY1485. (OCM1 cells: A-B; MUM-2B cells: C-D)

.


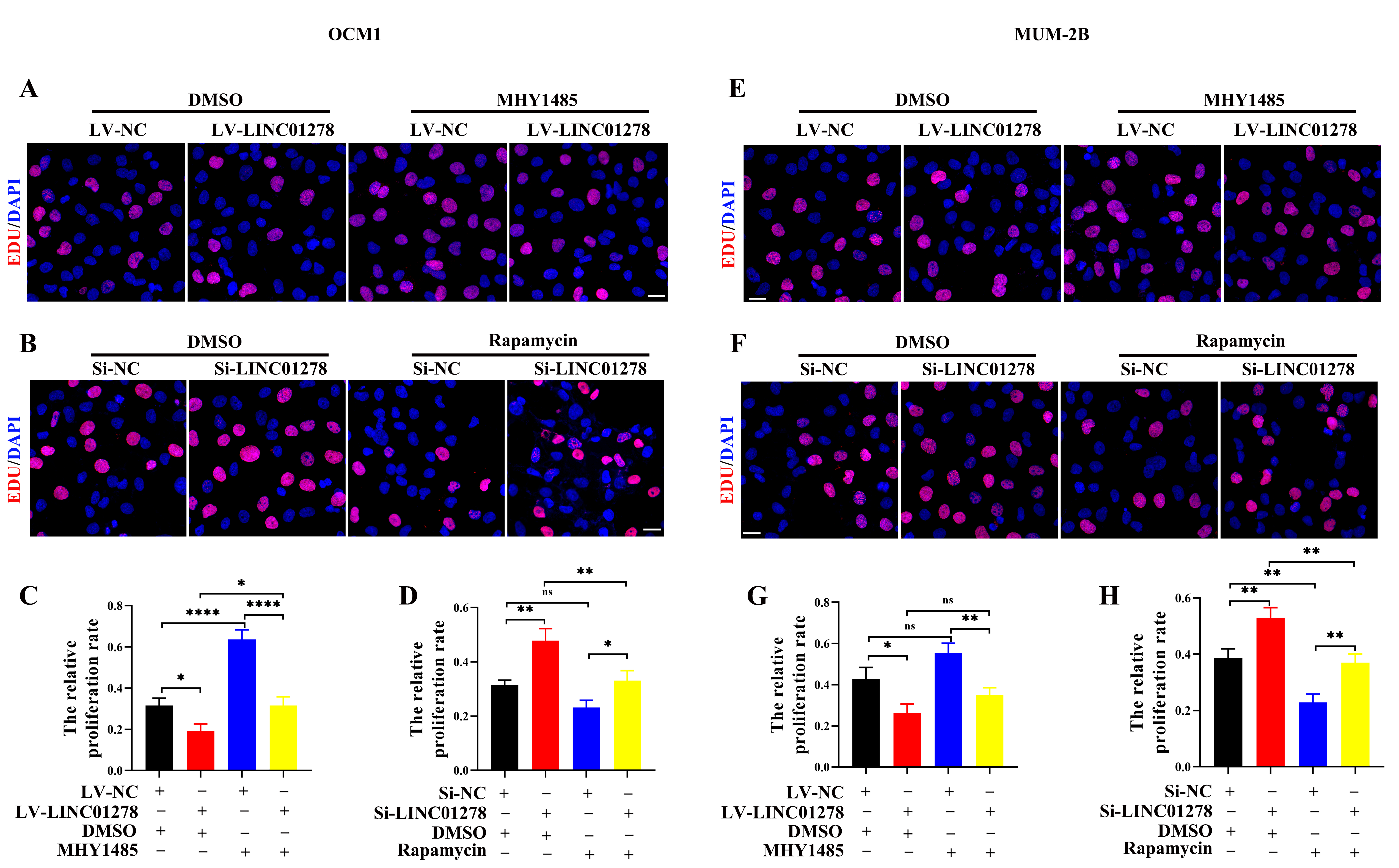


**Fig. S10 LINC01278 inhibits the proliferation of UM cells** **by suppressing the mTOR signalling pathway**.

**(A and E)** Proliferation analyses of the LV-NC group and LV-LINC01278 group after treatment with DMSO or MHY1485 by the EDU assay. **(C and D)** C is the statistical results of A and D is the statistical results of B. **(B and F)** Proliferation analyses of the Si-NC group and Si-LINC01278 group after treatment with DMSO or Rapamycin by the EDU assay. **(G and H)** G is the statistical results of E and H is the statistical results of F. (OCM1 cells: A-D; MUM-2B cells: E-H; scale bar: 60 µm; data are presented as the mean ± SD; n = 3; ns: no significant difference, *p < 0.05, **p < 0.01, ***p < 0.001, **** p < 0.0001).


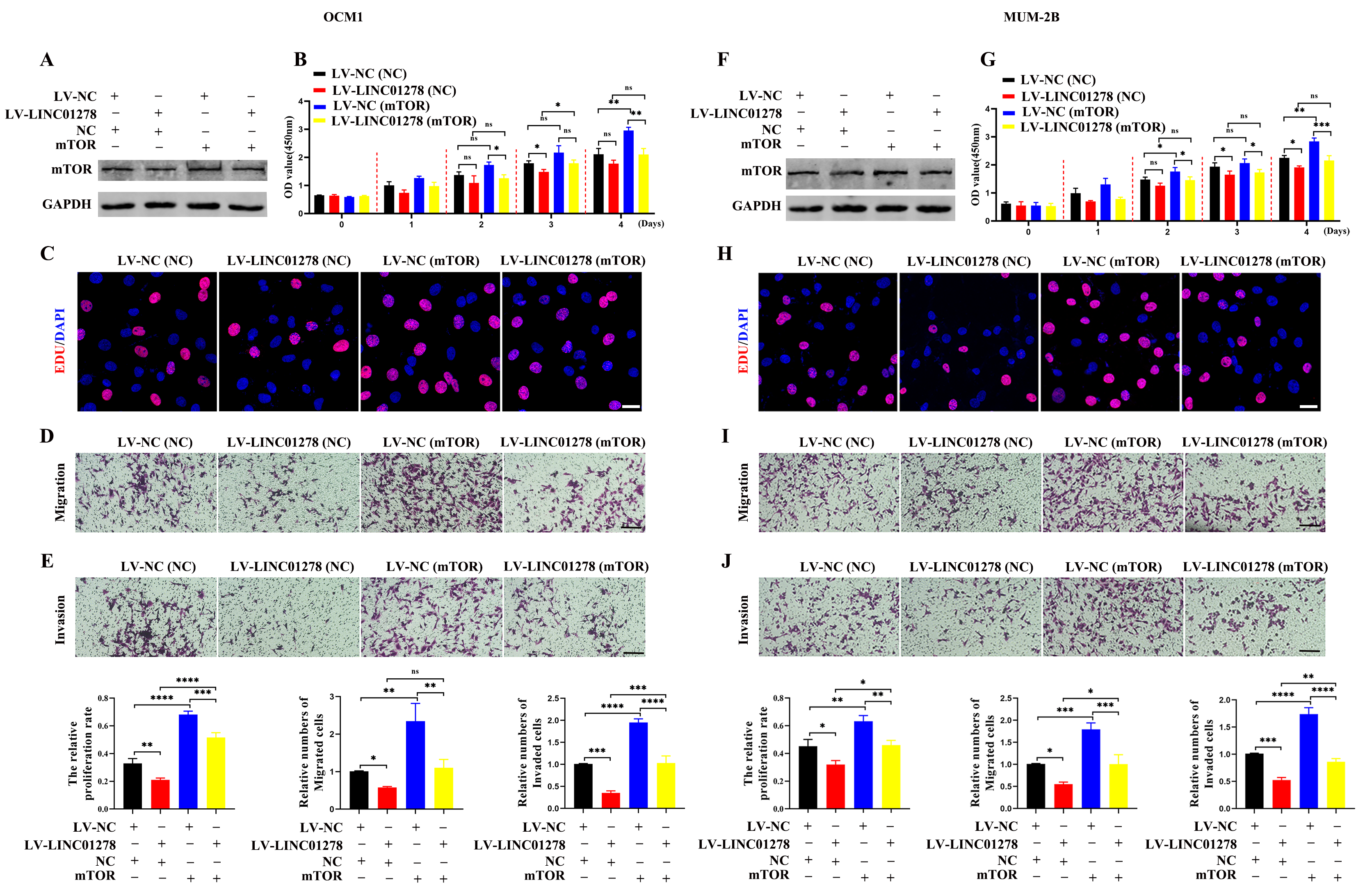


**Fig. S11** **LINC01278 inhibits** **UM progression** **by suppressing mTOR protein expression.**

**(A and F)** Western blot analysis of mTOR in the LV-NC group and LV-LINC01278 group after overexpressing mTOR. **(B and G)** Proliferation analyses of the LV-NC group and LV-LINC01278 group after overexpressing mTOR by CCK8. **(C and H)** Proliferation analyses of the LV-NC group and LV-LINC01278 group after overexpressing mTOR by the EDU assay. **(D and I)** Migration analyses of the LV-NC group and LV-LINC01278 group after overexpressing mTOR. **(E and J)** Invasion analyses of the LV-NC group and LV-LINC01278 group after overexpressing mTOR. (OCM1 cells: A-E; MUM-2B cells: F-J; scale bar: 60 µm and 100 µm; data are presented as the mean ± SD; n = 3; ns: no significant difference, *p < 0.05, **p < 0.01, ***p < 0.001, **** p < 0.0001).

| **characteristics** | **TCGA-UM (n=80)** |
| --- | --- |
| **Age (years)** |  |
| <65 | 45 (56.25%) |
| ≥65 | 35 (43.75%) |
| **Gender** |  |
| Female | 35 (43.75%) |
| Male | 45 (56.25%) |
| **Stage** |  |
| I | 0 (0%) |
| II | 39 (48.75%) |
| III | 36 (45%) |
| IV | 4 (5%) |
| Unknown | 1 (1.25%) |
| **T classification** |  |
| T1 | 0 (0%) |
| T2 | 14 (17.5%) |
| T3 | 32 (40%) |
| T4 | 34 (42.5%) |
| **M classification** |  |
| M0 | 51 (63.75%) |
| M1 | 4 (5%) |
| Unknown | 25 (31.25%) |

**Table S1 Clinical characteristics of UM patients involved in the research.**
